# FuFangChangTai Decoction Activates Macrophages via Inducing Autophagy

**DOI:** 10.1155/2019/5657035

**Published:** 2019-06-12

**Authors:** Lingchang Li, Haiyan Wang, Jun Qian, Guoli Wei, Rong Ding, Canhong Hu, Dong Fang, Ziyu Jiang, Lei Bi, Jie Song, Jun Ma, Fengxia Qin, Xiaofei Huang, Meng Cao, Jiege Huo

**Affiliations:** ^1^Affiliated Hospital of Integrated Traditional Chinese and Western Medicine, Nanjing University of Chinese Medicine, Nanjing, Jiangsu, China; ^2^Nanjing University of Chinese Medicine, Nanjing, Jiangsu, China; ^3^Xuzhou Tongshan District Hospital of Traditional Chinese Medicine, Xuzhou, Jiangsu, China; ^4^Zhenjiang Hospital of Chinese Traditional and Western Medicine, Zhenjiang, Jiangsu, China; ^5^Huaian Affiliated Hospital of Nanjing University of Chinese Medicine, Huaian, Jiangsu, China

## Abstract

The traditional Chinese medicine decoction FuFangChangTai (FFCT) has been used in the therapy of colon cancer clinically, yielding alleviated toxicity and enhanced immunity. In our previous study, FFCT exerted its antitumor activity not only by inducing apoptosis but also by activating autophagy to eliminate tumor cells. However, its mechanism is not well understood. The purpose of this study was to investigate the relationship between macrophages activation and FFCT-induced autophagy. Results showed that FFCT could induce autophagy in colon cancer, as demonstrated by increased level of intracellular autophagy marker LC3 II in CT26.WT cells by fluorescence microscope and western blot assay. FFCT also facilitated numbers of vesicular bodies with bilayer membrane in CT26.WT cells, which were indicative of autophagosomes formation. Autophagosomes secreted by FFCT-treated CT26.WT cells can activate M1 type macrophages, accompanied with increased expression of costimulatory molecules CD86 and CD40 on the surface of RAW264.7 cells, and more inflammatory cytokines secretion, such as TNF-*α*, IL-6, MCP-1, and IL-1*β*. mRNA expressions of M2 macrophages markers, such as IL-10, CD206, Arg-1, and FIZZ-1, were downregulated. And this process helps regulate the polarization of macrophages and promote the immune response. These findings support a mechanism of FFCT-induced autophagy and provide novel evidence demonstrating that macrophages are involved in FFCT-induced autophagy progression.

## 1. Introduction

Traditional Chinese medicine (TCM) plays an important role in the comprehensive treatment of colon cancer in China with the effects of inhibiting cell proliferation, promoting cell apoptosis [1], preventing tumor metastasis [2], blocking angiogenesis [3], reversing multidrug resistance [4], and alleviating the side effects of radiotherapy and chemotherapy [5].

The TCM decoction FuFangChangTai (FFCT) is composed of ginseng, astragalus mongholicus, semen coicis, Chinese actinidia root, kuh-seng, and fiveleaf akebia fruit. The results of a small clinical sample study on the treatment of colorectal cancer showed that FFCT combined with FOLFOX4 regimen alleviated the toxicity, compared with the single chemotherapy regimen [6]. The clinical symptoms of patients were significantly improved. The levels of immunological markers represented by T-lymphocyte typing were significantly increased, and the incidence of leukopenia was considerably decreased [6]. Our previous experiments showed that FFCT had synergistic effect with chemotherapy in inhibiting tumor weight* in vivo* and could obviously improve organ atrophy induced by cyclophosphamide chemotherapy in H22 tumor-bearing mice. The serum hemolysin level of H22 tumor-bearing mice was increased, and the decrease of serum hemolysin content induced by chemotherapy was obviously restored. The humoral immune ability of H22 tumor-bearing mice was improved by increasing the thymus and spleen index, which reflects immunologic function of mice [7].

FFCT could also inhibit the proliferation of human colon cancer cell line SW480 by blocking G0/G1 and G2/M cell phases in a dose-dependent and time-dependent manner [8]. And the apoptosis and necrosis of colon cancer cells were amplified meaningfully, also in a dose-dependent and time-dependent manner. The changes of early apoptosis suggested that apoptosis was one of the mechanisms of FFCT inhibiting the growth of colon cells. Caspase-3 was an important pathway for FFCT to promote apoptosis of SW480 cells [9]. The changes of late apoptotic and necrotic rates suggested that FFCT's inhibition on tumor cell growth was also mediated by other pathways. Furthermore, a large number of autophagy bubbles were observed in SW480 cells treated with FFCT. As demonstrated in immunofluorescence staining and western blot assay, it was confirmed that FFCT could induce autophagy in SW480 cells.

In response to stress, autophagy exhibits defensive functions, contributing to cell survival in adverse conditions, and conversely it has also been involved in cell death. The morphology of autophagosome is characteristic of a double membrane as an integral part. Autophagosomes fuse with lysosomes to degrade substances contained within autophagosomes [10]. Activation of autophagy in macrophages causes phagosomes to mature into autolysosomes. Autophagy is a highly conserved process, involving forming autophagosomes that deliver macromolecules and whole organelles or intracellular pathogens to lysosomes for degradation [11]. LC3, the mammalian homologue of Atg8, and its lipidated form, LC3-II, are present on autophagosomes during canonical autophagy [12]. Macroautophagy, a homeostatic process that shuttles cytoplasmic constituents into endosomal and lysosomal compartments, has recently been shown to deliver antigens for presentation on major histocompatibility complex (MHC) class II. Substrates of autophagy can be loaded onto MHC class II for CD^4+^ T cell recognition [13].

The therapeutic effect of FFCT on colorectal cancer has been proved in clinical and experimental research; however, its specific mechanism remains to be further explored. This paper aims to discuss the relationship between autophagy induced by FFCT and the activation of macrophages and its antitumor immune response.

## 2. Materials and Methods

### 2.1. Materials

FFCT was composed of six ingredients, and its components and amounts (g) were listed as follows: 10 g ginseng, 15 g astragalus mongholicus, 20 g semen coicis, 15 g Chinese actinidia root, 6 g kuh-seng, and 10 g fiveleaf akebia fruit which were all purchased from Chinese Pharmacy, Jiangsu Provincial Hospital of Integrated Chinese and Western Medicine (Nanjing, China). The mouse colon cancer cells CT26.WT and mouse macrophages RAW264.7 were preserved in Molecular Biology Laboratory of Jiangsu Institutes of TCM (Nanjing, China) which were purchased from American Type Culture Collection (ATCC, Manassas, VA). Anti-LC3 I and anti-LC3 II antibodies were purchased from Cell Signaling Technology (Danvers, MA). Anti-*β*-actin was purchased from Santa Cruz Biotechnology (Santa Cruz, CA). DAPI, Lipofectamine 2000, and rapamycin were bought from Sigma-Aldrich (Shanghai, China). Opti-MEM, RPMI 1640 medium, and DMEM medium were purchased from Keygen Biotech (Nanjing, China). BCA assay was purchased from Beyotime Biotechnology (Haimen, China). All of the other reagents used in this study were of an analytical grade.

### 2.2. FFCT Extraction

All the TCM listed above were mixed together, soaked in water, and extracted by 100°C water for 2 h. The extract was then filtered, rotary evaporated for concentration, and lyophilized to make FFCT powder. The product was stored at -80°C until use.

### 2.3. Cell Culture

The CT26.WT cells were cultured in RPMI 1640 medium supplemented with 10% fetal bovine serum (FBS) from Zhejiang Tianhang Biotechnology (Hangzhou, China) and 100 *μ*g/mL antibiotics (100 U/mL penicillin and 100 *μ*g/mL streptomycin) in a 5% CO_2_ humidified incubator at 37°C. The RAW264.7 cells were cultured in DMEM supplemented with 10% FBS and 100 *μ*g/mL antibiotics (100 U/mL penicillin and 100 *μ*g/mL streptomycin) in a 5% CO_2_ humidified incubator at 37°C.

### 2.4. Cell Viability Assay

CT26.WT cells at logarithmic growth stage were collected and resuspended to 10^4^ cells/100 *μ*L cell suspension and then inoculated in 96-well plate. 10, 20, 30, and 40 mg/mL FFCT were added to cells, respectively, in 96-well plate while the control group was without any treatment. After culture for 48 h, the medium in each well was substituted with 20 *μ*L MTT solution (5 mg/mL) and incubated at 37°C for 4 h. The MTT solution was displaced with 150 *μ*L DMSO. The absorbance value was measured at 570 nm wavelength by Microplate Spectrophotometer (Bio-Tek Epoch 2).

### 2.5. Effects of FFCT on the Distribution of Autophagy Marker Protein LC3 in CT26.WT Cells

eGFP plasmids were transferred into CT26.WT cells by lipofectamine 2000 in Opti-MEM and incubated at 37°C for 6 h and then displaced with complete medium and cultured for further 18-72 h. After screening with gradient concentrations of G418, CT26.WT cell lines with stable expression eGFP were obtained with 20 *μ*g/mL G418. 10, 20, 30, and 40 mg/mL FFCT and 50 nM rapamycin (positive control group) were added to eGFP-CT26.WT cell, respectively, in 6-well plate while the negative control group was without any treatment and was incubated at 37°C for 48 h. The distribution of green fluorescence was observed under fluorescence microscope.

### 2.6. Transmission Electron Microscopy

Transmission electron microscopy (TEM) was used to confirm whether the cytosolic vesicular particles observed under the microscope are related to autophagy as previously described [14].

### 2.7. BCA Protein Determination

CT26.WT cells at logarithmic growth stage were collected and resuspended to 5×10^4^ cells/mL cell suspension and then inoculated to 6-well plate. 10, 20, 30, and 40 mg/mL FFCT and 50 nM rapamycin (positive control group) were added to cells, respectively, in 6-well plate while the negative control group was without any treatment. Cultured for 48 h, the medium was discarded and cell lysis buffer containing 100 mM phenylmethanesulfonyl fluoride was added to cells and lysing for 30 min on ice. Then we collected the mixture and centrifuged it at 4°C, 12000 rpm for 30 min. The protein sample 10-20 times was diluted with deionized water and the protein content was analyzed with BCA protein assay according to the protocol.

### 2.8. Western Blot

In order to further confirm whether the cytoplasmic vesicular substance observed under inverted microscope is related to autophagy, vesicular substance was extracted. The expression of LC3-II in the extracted vesicles was identified by Western blot. Protein samples of CT26.WT cells treated with different concentration of FFCT and rapamycin were also analyzed with western blot to determine the expression level of LC3 I and LC3 II as previously described [15].

### 2.9. Flow Cytometry

The autophages extracted from FFCT-treated CT26.WT cells were mixed with fluorochrome: 5, 6-carboxyfluorescein diacetate succinimidyl ester (CFSE) (Biolegend, San Diego, CA) to a final concentration of 5 mM, incubated at 37°C for 15 min in dark, and terminated by adding precooled complete medium, and then we kept the mixture in dark for 10 min at room temperature. Extra CFSE was quenched with precooled FBS and centrifuged at 2000 rpm for 5 minutes. The supernatant was discarded and washed twice with precooled PBS. The autophages were adjusted to 5 mM with PBS, and then the autophages were added to RAW264.7 cells and negative control group was without any treatment and was cultured at 37°C for 48 h. Cells of the both groups were collected and washed twice with PBS and then suspended with 500 *μ*L 4% paraformaldehyde for flow cytometry (Beckman Coulter) [16].

### 2.10. Phagocytosis of RAW264.7 Cells on CFSE-Labeled Autophagic Bodies Secreted by CT26.WT Cells via Laser Confocal Microscope

The autophagic vacuole secreted by CT26.WT cells was collected and labeled with CFSE. The autophages were then added to RAW264.7 cells and negative control group was without any treatment and was cultured at 37°C for 6 h. The uptake of CFSE-labeled autophagy by macrophages was observed with laser confocal microscopy (Olympus) [17].

### 2.11. ELISA and qPCR

The autophages were added to RAW264.7 cells and negative control group was without any treatment and was cultured at 37°C for 6 h. The supernatant of macrophages was collected to test the content of TNF-*α* and IL-6 with ELISA. mRNA was extracted to examine changes of mRNA expression of TNF-*α*, IL-6, MCP-1, IL-1*β*, IL-10, CD206, Arg-1, and FIZZ-1 in macrophages with qPCR. After FFCT treatment, cells were subjected to total RNA extraction. QPCR was performed on BIO-RAD iQ5TM real-time PCR system with SYBR Green Real-time PCR Master MIX: one cycle of 95°C for 30 s and 40 cycles of 95°C for 5 s, 55°C for 10 s, and 72°C for 15 s. Primers sequences are listed in Table 1.

### 2.12. Statistical Analysis

The intergroup variation between various groups was measured by one-way analysis of variance (ANOVA) followed by Dunnett's multiple comparison test, and the comparisons between two groups were conducted by unpaired Student's* t*-test (SPSS Version 13).* P* < 0.05 was considered to be statistically significant.

## 3. Results

### 3.1. FFCT Induced Autophagy in Colon Cancer

**Figure 1:**
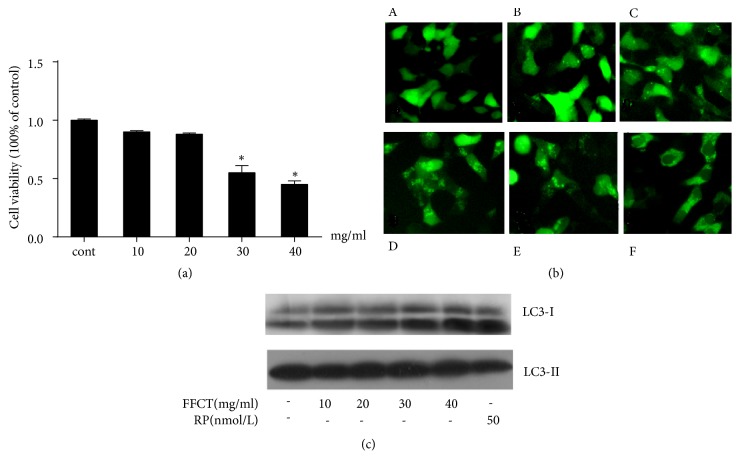
FFCT induces autophagy in colon cancer: (a) effect of FFCT on the viability of CT26.WT cells. ^*∗*^* P *< 0.05 versus the control group; (b) formation of intracellular autophagy marker eGFP-LC3 II on CT26.WT cells by fluorescence microscope. A. Negative control, B. CT26.WT cells were treated with FFCT (10 mg/mL, 48 h), C. FFCT (20 mg/mL, 48 h), D. FFCT (30 mg/mL, 48 h), E. FFCT (40 mg/mL, 48 h), F. positive control, CT26.WT cells were treated with rapamycin (50 nM, 48 h); (c) effect of FFCT on the expression of LC3 protein in CT26.WT cells after 48 h by western blot assay.

In order to establish an effective experimental model, it is necessary to analyze the effect of FFCT on the viability of colon cancer CT26.WT cells, so as to eliminate the cytotoxicity of FFCT on colon cancer cells. CT26.WT cells were treated with FFCT (10, 20, 30, and 40 mg/mL), respectively. After incubation with 10 and 20 mg/mL FFCT for 48 h, no significant changes were found in cell activity and cytotoxicity (*P *> 0.05). But 30 and 40 mg/mL FFCT demonstrated obvious cytotoxicity in CT26.WT cells (*P *< 0.01) (Figure 1(a)). The results showed that FFCT in the range of 10-20 mg/mL could fulfill the requirement of the follow-up experiment.

The presence of eGFP-LC3 was used as an indicator of autophagosome formation and the occurrence of autophagy [2]. It showed that eGFP-LC3 spots in FFCT-treated CT26.WT cells and the positive control rapamycin-treated CT26.WT cells were higher than that in the negative control (Figure 1(b)). Western blot assay was performed to verify whether FFCT introduces autophagy. As expected, the expression of LC3 II protein was higher in both rapamycin and FFCT groups. Different concentrations of FFCT (10, 20, 30, and 40 mg/mL) and 50 nM rapamycin induced autophagy in CT26.WT cells (Figure 1(c)).

### 3.2. FFCT Induced Autophagosomes in Colon Cancer Cells

**Figure 2:**
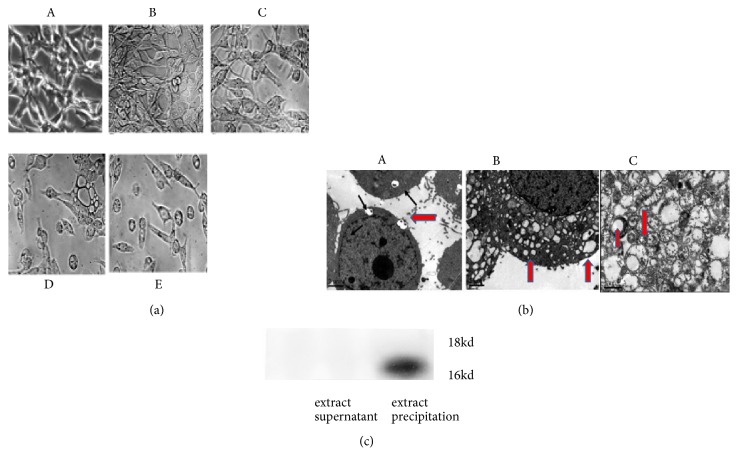
Extraction and identification of autophagy secreted by CT26.WT cells induced by FFCT: (a) morphological changes of CT26.WT cells treated by FFCT by microscope. A. Negative control, B. CT26.WT cells were treated with FFCT (10 mg/mL, 48 h), C. FFCT (20 mg/mL, 48 h), D. FFCT (30 mg/mL, 48 h), E. FFCT (40 mg/mL, 48 h); (b) observation of structural changes of CT26.WT cells treated with FFCT by TEM. A and B. Vesicular granular structure treated by FFCT, C. autophagosome with double-layer membrane structure that encapsulates part of the organelle; (c) expression of autophagy marker protein LC3-II in extract by western blot.

The morphological changes of CT26.WT cells were observed under inverted microscope after treatment of FFCT at different concentrations (10, 20, 30, and 40 mg/mL) for 48 h. The cells in the negative control group had good adherence and regular morphology. After treatment with FFCT for 48 h, significant morphological changes were observed in the cytoplasm, and the vesicles in the cytoplasm of the cells showed varying sizes and irregular shapes. And with the increment of FFCT concentration, the cytosolic vesicles increased gradually (Figure 2(a)).

TEM showed that there were a large number of vesicular bodies with bilayer membrane structure and varying sizes and residual organelles in some of them in the cytoplasm of the FFCT-treated group. The structural characteristics of the cells were similar to those of autophagy (Figure 2(b)). The expression of LC3-II was identified by western blot. As shown in Figure 2(c), the vesicles extract contained a high level of LC3-II, which confirmed that autophagosomes were secreted by tumor cells treated with FFCT.

### 3.3. Macrophages Can Recognize and Uptake Autophagosomes Secreted by FFCT-Treated Colon Cancer Cells

**Figure 3:**
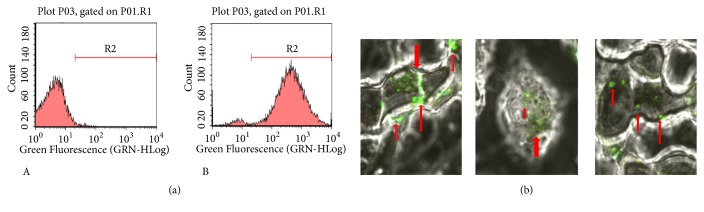
Macrophages uptake FFCT-induced autophagosomes secreted by colon cancer cells: (a) identification and uptake of CFSE-labeled autophagy in RAW264.7 by flow cytometry. A. RAW264.7 as a negative control group, B. CSFE-labeled autophagosomes extracted from FFCT-treated CT26.WT cells were added in RAW264.7 cells; (b) study on the recognition and uptake of CFSE-labeled autophagy in RAW264.7 by confocal laser. The green fluorescent spots in the image were CFSE-labeled autophagosome.

We used 5, 6-carboxyfluorescein diacetate succinimidyl ester (CFSE), a dye that can label living cells with fluorescence. The results showed that the FFCT-induced autophagosomes secreted by CT26.WT could be recognized by macrophage RAW264.7 and phagocytic proliferation (Figure 3(a)). Laser confocal microscope results showed that obvious green fluorescence spots could be observed in RAW264.7 cells after incubating with the extracted autophagosomes for 6 h, which indicated that RAW264.7 cells had strong uptake ability to CFSE-labeled autophagosomes (Figure 3(b)).

### 3.4. Autophagosomes Secreted by FFCT-Treated Colon Cancer Cells Can Activate M1 Type Macrophages

**Figure 4:**
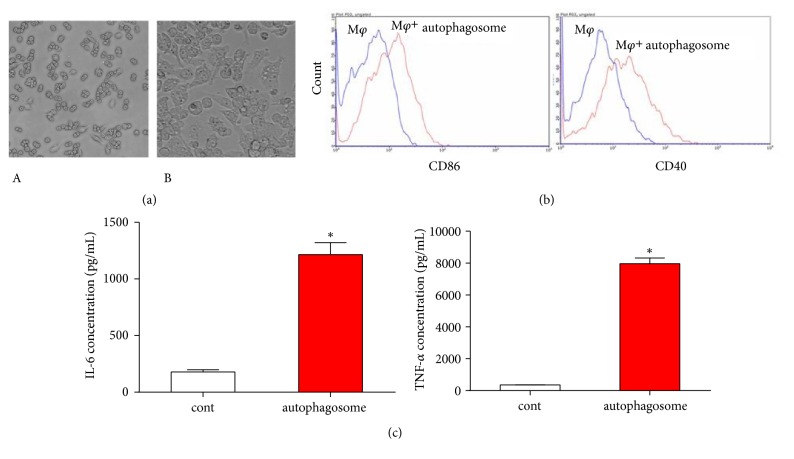
Activation of macrophages induced by FFCT in colon cancer cells: (a) morphology of autophagosome in RAW264.7 cells. A. Negative control, B. RAW264.7 cells were added with CSFE-labeled autophagosome extracted from FFCT-treated CT26.WT cells; (b) the autophagosome produced by FFCT-treated CT26.WT cells facilitated the expression of CD86 and CD40 on the surface of RAW264.7 cells; (c) cytokines expression in the supernatant of RAW264.7 cells cultured with autophagosomes for 48 h (TNF-*α* and IL-6). ^*∗*^* P *< 0.05.

Mouse macrophages RAW264.7 were round or elliptical in unstimulated state, with clear boundary, good adhesion, and few protrusions (Figure 4(a)). After stimulating RAW264.7 cells with autophagosomes secreted from FFCT-treated CT26.WT cells, the volume of macrophages was significantly larger than that of the blank group, and there were irregular vesicles in the cytoplasm of the macrophages, and more pseudopod formation was observed as well (Figure 4(b)).

The expression of membrane surface molecules and costimulatory molecules is an important indicator to determine whether macrophages are polarized or not. We used flow cytometry to examine the main surface markers of M1 macrophages including CD86 and CD40. Moreover, the expression of costimulatory molecules on macrophages was closely related to the ability of T lymphocytes to present antigen-activated effects. The results showed that the expressions of costimulatory molecules CD86 and CD40 on the surface of RAW264.7 cells were upregulated after 48 h incubation with autophagy produced by FFCT-treated CT26.WT cells (Figure 4(b)).

Macrophages synthesize and secrete a variety of cytokines, which play an important role in the transmission of information in cells and improve the immune function of organism. TNF-*α* and IL-6 are two typical proinflammatory cytokines, mainly secreted by activated macrophages. ELISA was used to investigate the effect of autophagosomes on the production of TNF-*α* and IL-6. TNF-*α* and IL-6 cytokines in the supernatant of RAW264.7 cells cultured with autophagosomes for 48 h were upregulated, suggesting that RAW264.7 was activated by autophagosomes and cytokines could probably affect immune effector cells and further enhance the phagocytosis ability of macrophages to kill pathogens (Figure 4(c)).

**Figure 5:**
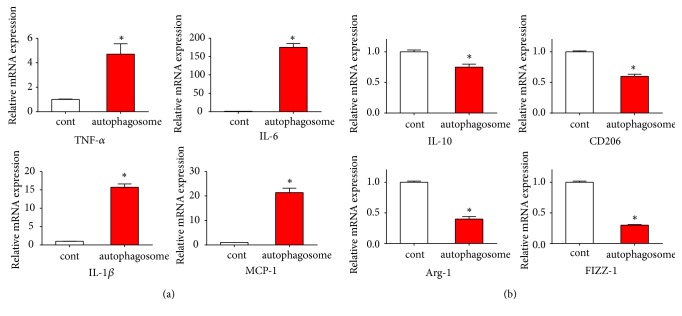
Autophagosomes secreted by FFCT-treated CT26.WT cells can promote the polarization of macrophages from M2 type to M1 type: (a) mRNA expression of TNF-*ɑ*, IL-6, MCP-1, and IL-1*β* when cultured with autophagosomes. ^*∗*^* P *< 0.05. (b) mRNA expression of IL-10, CD206, Arg-1, and FIZZ-1 when cultured with autophagosomes. ^*∗*^* P *< 0.05.

We next examined mRNA expression of TNF-*α*, IL-6, MCP-1, and IL-1*β* in macrophages with qPCR. The results showed that, when cultured with autophagosomes produced by FFCT-induced CT26.WT cells for 48 h, the expressions of TNF-*α*, IL-6, MCP-1, and IL-1*β* were all upregulated (Figure 5(a)). Macrophages display a spectrum of phenotypes between two extremes identified as antitumor M1 and reparative M2 macrophages, characterized by the expression of specific cell surface markers and the secretion of different cytokines. In order to fully demonstrate the polarization of macrophages towards M1 type by FFCT, we carried out qPCR experiments to detect the mRNA expression of M2-related genes, such as IL-10, CD206, Arg-1, and FIZZ-1. As expected, mRNA expressions of IL-10, CD206, Arg-1, and FIZZ-1 in macrophages were downregulated after 48 h of autophagosomes treatment (10 *μ*g/mL), suggesting that autophagosomes secreted by FFCT-treated CT26.WT cells can promote the polarization of macrophages from M2 type to M1 type (Figure 5(b)).

## 4. Discussion

Autophagy is a biological process during which cells use their own lysosomes to degrade damaged, denatured, or senescent proteins and organelles. Autophagy plays an important role in tumorigenesis and development. However, autophagy displays a dual role in tumor development [18–20]. On one hand, autophagy can protect tumor cells under stress by removing pathogens, damaged organelles, or misfolded proteins [21]. Autophagy can promote their proliferation and maintain their growth. On the other hand, autophagy promotes cell death through oxidative stress and DNA damage. Excessive autophagy leads to excessive consumption of cells and induces death, also known as type II programmed death [22]. Some research showed that, when the expression of the autophagy inducing gene beclin was downregulated, the degree of autophagy was positively correlated with the occurrence of tumor [23–25]. Therefore, it is of significance to search for drugs that can promote autophagy-induced death of tumor cells.

The homologue of mammalian ATG8 is LC3, and the C-terminal is cut by cysteine protease ATG4 and activated by ATG7 (E1) and ATG3 (E2), which binds to phosphatidylethanolamine on the membrane [26]. LC3 exists in the form of LC3-I and LC3-II. LC3 changed from the free state of LC3-I to a stable position on the membrane of autophagy, so the expression of LC3-II indicates the existence of autophagy [27]. LC3-II can also be used to detect the level of autophagy expression by labeling the N-terminal of LC3 with fluorescent proteins such as GFP [28]. The localization analysis of autophagy serves as an important method for detecting autophagy activity in cells [29]. eGFP-LC3 indicates that it produces autophagy. During autophagy in mammalian cells, the content of LC3-II and the transformation of LC3-I to LC3-II rise meaningfully. The high expression of autophagy marker protein LC3II was confirmed by western blot. The results in this study showed that there was no LC3-II protein in colon cancer CT26.WT cells in blank control group, and the expression of LC3-II in CT26.WT cells was dose-dependently enlarged after treatment with FFCT for 48 h. The cytosolic vesicles produced by CT26.WT cells which were previously treated with FFCT were observed under microscope. The vesicle numbers increased gradually as the concentration of FFCT upsurges. By observing the ultrastructure of vesicles with transmission electron microscope, we found that the autophagy is a double-layer membrane structure.

Macrophages are important cellular components of the organism immune system [30]. However, antigen-presenting can kill tumor cells only when macrophages are activated [31–33]. The expression of surface costimulatory molecules is an important index to identify the polarization of macrophages [34, 35]. Activated macrophages express MHC-II molecules and costimulatory molecules such as CD80, CD86, and CD40 [36]. The costimulatory molecules interact with the receptor molecules on the surface of T cells [37]. In this study, flow cytometry was used to detect the effect of FFCT-induced autophagy secreted by CT26.WT cells on the surface molecules CD80, CD86, and CD40 of macrophages. The results showed that autophagy could upregulate the expression of costimulatory molecules on macrophages and affect the antigen presentation of macrophages.

Activated M1 macrophages have potent ability to synthesize and secrete cytokines and complement components and participate in immune response [38, 39], such as TNF-*α*, IL-6, MCP-1, and IL-1*β*. TNF-*α* has anti-inflammation effects, inhibiting or killing tumor cells [40, 41]. IL-6 can activate T cells and B cells and then promote the specific immune response [42, 43]. It plays an important role in tumorigenesis and development [44]. IL-1*β* is an essential medium for macrophages to activate T cells, which can increase the expression of MHC-II antigen on T cells and induce cytotoxic T cells [45]. MCP-1 is a chemotactic cytokine that can make T cells and NK cells chemotactic and enhances immunity [46]. In this study, the above surface costimulatory molecules and cytokines were selected to elucidate that the autophagy secreted by FFCT on CT26.WT cells in the course of activated macrophage antitumor immune response. The cytosolic vesicles produced by FFCT-treated CT26.WT cells secreted TNF-*α*, IL-6, MCP-1, and IL-1*β*, which promotes the activation of the macrophages.

M1 macrophages play an important role in inflammatory response and antitumor immunity. While M2 macrophages are immunosuppressive cells and promote angiogenesis, tissue repair, and reconstruction, M2 macrophages support Th2-associated effector functions and play a key role in regulating T cell functioning. Their antigen presenting ability is weak. And CD206 (mannose receptor), Arg-1, and FIZZ-1 are highly expressed in M2 macrophages. By releasing immunosuppressive factors such as IL-10, M2 macrophages can inhibit the immune response of T lymphocyte and NK cell and decrease the immune response of T lymphocyte and NK cell. Retnla/Relma/FIZZ1 is a member of a family of cysteine-rich secreted proteins, referred to “resistin-like molecules” or “found in inflammatory zone” originally found in allergic inflammation. mRNA expressions of IL-10, CD206, Arg-1, and FIZZ-1 in macrophages were downregulated after 48 h of autophagosomes treatment (10 *μ*g/mL), suggesting that autophagosomes secreted by FFCT-treated CT26.WT cells can promote the polarization of macrophages from M2 type to M1 type.

## 5. Conclusion

This study is helpful to elucidate the antitumor effect of FFCT as an immunomodulator on colorectal cancer cells and M1 type macrophages. FFCT can induce the autophagy of colon cancer CT26.WT cells, which helps regulate the polarization of macrophages and helps promote the immune response. The research contributes to the development and utilization of FFCT and provides a new strategy for anticancer drugs screening. But whether the autophagy induced by FFCT has crucial effect with its antitumor effect needs further study.

## Figures and Tables

**Table 1:** Sequences of primers used in real-time polymerase chain reaction.

Gene	Primer sequences (5′-3′)	
TNF-*α*	Forward	GTGTCCCAACATTCATATTGTCAGT
	Reverse	TGGGAAGAGAAACCAGGAGA

IL-6	Forward	GTCTTGGCCGAGGACTAAGG
	Reverse	TACTCGGCAAACCTAGTGCG

IL-1*β*	Forward	TGGGATAGGGCCTCTCTTGC
	Reverse	CCATGGAATCCGTGTCTTCCT

MCP-1	Forward	CCCCAGTCACCTGCTGTTAT
	Reverse	TGGAATCCTGAACCCACTTC

IL-10	Forward	CCAAGCCTTATCGGAAATGA
	Reverse	TTCACAGGGGAGAAATCG

Arg-1	Forward	CAAGACAGGGCTCCTTTCAG
	Reverse	GTAGTCAGTCCCTGGCTTATGG

FIZZ-1	Forward	TCCCAGTGAATACTGATGAGA
	Reverse	CCACTCTGGATCTCCCAAGA

CD206	Forward	CAGGTGTGGGCTCAGGTAGT
	Reverse	TGTGGTGAGCTGAAAGGTGA

GAPDH	Forward	GTCTTCACCACCATGGAG
	Reverse	CCAAAGTTGTCATGGATGACC

## Data Availability

The data used to support the findings of this study are available from the corresponding author upon request.

## Abbreviations

FFCT: FuFangChangTai
TCM: Traditional Chinese medicine
TEM: Transmission electron microscopy
TNF-*α*: Tumor necrosis factor-*α*
IL-6: Interleukin-6
MCP-1: Monocyte chemotactic protein-1
IL-1*β*: Interleukin-1*β*
PBS: Phosphate buffer saline
CFSE: 5, 6-Carboxyfluorescein diacetate succinimidyl ester
IL-10: Interleukin-10
FIZZ-1: Resistin-like molecule alpha/Retnla
Arg-1: Type-1 arginase.
